# ‘Cleanse or Die’: British Naval Hygiene in the Age of Steam, 1840–1900

**DOI:** 10.1017/mdh.2018.3

**Published:** 2018-04

**Authors:** Elise Juzda Smith

**Affiliations:** Faculty of History, University of Warwick, Humanities Building, University Road, Coventry CV4 7AL, UK

**Keywords:** Royal Navy, Naval medicine, Hygiene, Sanitation, Ventilation, Etiology

## Abstract

This article focuses on the consolidation of naval hygiene practices during the Victorian era, a period of profound medical change that coincided with the fleet’s transition from sail to steam. The ironclads of the mid- to late- nineteenth century offered ample opportunities to improve preventive medicine at sea, and surgeons capitalised on new steam technologies to provide cleaner, dryer, and airier surroundings below decks. Such efforts reflected the sanitarian idealism of naval medicine in this period, inherited from the eighteenth-century pioneers of the discipline. Yet, despite the scientific thrust of Victorian naval medicine, with its emphasis on collecting measurements and collating statistics, consensus about the causes of disease eluded practitioners. It proved almost impossible to eradicate sickness at sea, and the enclosed nature of naval vessels showed the limitations – rather than the promise – of attempting to enforce absolute environmental controls. Nonetheless, sanitarian ideology prevailed throughout the steam age, and the hygienic reforms enacted throughout the fleet showed some of the same successes that attended the public health movement on land. It was thus despite shifting ideas about disease and new methods of investigation that naval medicine remained wedded to its sanitarian roots until the close of the nineteenth century.

In the nineteenth century, the Royal Navy enjoyed a particular reputation for cleanliness. For officers and sailors, the continuous washing and fumigating of the decks may have been a ritual of order and discipline, but for the surgeons serving with them, it was upheld as a life-saving exigency. When the surgeon Norman Chevers half-jokingly suggested in 1846 that the Navy’s motto ought to be ‘cleanse or die!’, his views reflected the widespread belief amongst medical men that sanitary precautions were the key to preserving health afloat.^1^

From the late eighteenth century onwards, the challenges of instituting the principles of preventive medicine at sea revolved around maintaining a clean, dry, and well-ventilated environment within spaces that were invariably overcrowded, damp, and enclosed. The notorious insalubrity of ships remained axiomatic until the manpower requirements of warfare spurred efforts to minimise disease casualties. In Britain, the recommendations of maritime health authorities such as James Lind (1716–94), Thomas Trotter (1760–1832), and Gilbert Blane (1749–1834) led the Admiralty to institute reforms in every area of naval hygiene: sanitation and ventilation; dietary and drink allowances; clothing and bedding; exercise and rest.^2^ The benefits conferred by prioritising sanitary and dietary concerns were particularly shown during James Cook’s exploratory voyages of the 1770s, which boasted such low levels of mortality and morbidity that the value of preventive medicine was established beyond all doubt.

Yet, if naval hygiene was pioneered under sail, its precepts were most fully realised in the age of steam. As this article shows, technological advances in the nineteenth century vastly improved the potential for preventive medicine afloat. However, they also presented health problems of their own, challenging surgeons to overcome such hygienic hazards as the heat of the boilers, the damp of condensation on metal sidings, and the limited airflow to lower compartments. Innovative strategies for preserving the health of seafarers were constantly suggested and trialled, even while the rationales underpinning such measures were subjected to increased scrutiny – both from practical experience and from new etiological models that challenged the traditional link between sickness and the environment. Although Victorian naval surgeons believed that their ships might become fortresses against disease, their efforts to prevent epidemics often failed. This schism between theory and experience resulted in a conflicted system of medical practice in which preventive models continued to hold sway, even while their limitations were acknowledged.

While the emphasis placed on hygiene may not have entirely eradicated sickness, the Admiralty certainly used the conversion to steam to improve sanitary conditions. Their efforts raised levels of health and comfort in the service, and ultimately produced a decline in casualties during the second half of the nineteenth century.^3^ In this respect, the naval example anticipated advances made in the civilian sphere, where sanitary measures succeeded in raising the standard of living and limiting the spread of diseases whose origin remained a subject of debate.^4^ Yet, the parallels are not exact. While naval medical authorities claimed that hygienic principles were interchangeable whether on land or sea, the struggles of health authorities to institute preventive measures in the public sphere were not shared. Naval surgeons did not have to wrestle with officialdom for permission to implement their preventive strategies, nor did they have to contend with uncooperative citizens.^5^ Instead, the Navy’s command structure permitted them to enact their hygienic recommendations, and gave them nearly absolute control over the environment in which they served. It is precisely the self-contained nature of naval vessels that make them a critical model for considering how medical theory interacted with practical observations in a period of rapid technological change.

As recent scholarship has emphasised, nineteenth-century military medicine reflected the modernisation of warfare more broadly, as the principles of scientific management allowed for greater efficiency in the health provisions of armies and navies.^6^ Hygiene dominated this strategy: an ounce of prevention was worth several pounds of cure at a time when the therapeutic arsenal was comparatively limited. For Gilbert Blane, ships’ officers could far better ensure the health of crews through precautionary measures than surgeons could using the ‘falliable’ art of physic.^7^ As Katherine Foxhall and Tim Carter have shown for immigrant and merchant vessels, this ideal did not necessarily adapt to the management of shipboard health in the commercial sector, where skimming costs was often prioritised over seafarers’ welfare.^8^ But it was a necessity for the Royal Navy, whose manpower needs justified the expenses incurred in preserving lives.

In Admiralty instructions issued throughout the nineteenth century, surgeons were repeatedly reminded that ‘dryness, cleanliness, and ventilation’ were the preferred means of preventing and destroying the causes of disease.^9^ The wording of these instructions supported environmental theories of disease, which posited that noxious emanations from decomposing animal or vegetable matter were responsible for the propagation of infectious illnesses.^10^ These widely held views inspired sanitary reforms, although the competing contagionist model always had adherents arguing for the role of human contact in spreading sickness. Naval surgeons tended to favour the sanitarian position, although successive heads of the Medical Service sought to reframe this debate by rationalising the basis of maritime medicine. In extensive statistical reports compiled annually by the Admiralty, attempts were made to systematise all naval knowledge knowledge about disease.^11^ Surgeons were required to keep journals detailing every illness they encountered, and their findings were disseminated in annual health reports. Surgeons also published independent texts on naval hygiene, filling them with polemical advice about how best to preserve health at sea. These sources – by and for medical officers – reveal that naval medicine enacted broader disagreements in Britain around the origin and spread of infectious diseases. Because naval surgeons interpreted disease at sea, their ship ‘a little world within itself’, their experiences present a unique perspective on the state of epidemiological thought in the Victorian era.^12^ Their writings also show how technological advances could be used to stretch and test the limits of preventive care. This article thus examines how the ‘artificial’ living conditions aboard naval steamships inspired health reforms in the Victorian era, ultimately showing how beholden the sea service was to prevailing medical orthodoxy.

## 1 Preventive Medicine at Sea

If naval medicine in the nineteenth century was characterised by eclecticism, with surgeons facing a wide variety of ailments depending on the makeup and location of their vessels, then a belief in hygienic principles united them. Provided the causes of disease could be correctly identified, a timely implementation of precautionary measures would suffice to keep sickness at bay. It was well established by the early 1800s that masses of people in confined areas were particularly prone to ill health, and that shipboard epidemics were difficult to contain given the limited space and resources available to surgeons. Believing that counteracting the causes of disease would always ‘surpass in importance the means curative’, maritime medical texts consequently prioritised prophylaxis.^13^

The term ‘hygiene’ in this period broadly referred to all measures of preserving health. Specific preventatives such as quinine for malaria, citrus juices for scurvy, and smallpox vaccination had shown their value for particular diseases, but environmental management was heralded as the universal panacea against infection. Spaces that were light, dry, temperate, and airy were considered the most salubrious, but were almost impossible to create aboard ship. Nonetheless, the objective of Victorian naval hygiene was to maintain an ideal environment, and to manage every other factor affecting health: from ‘good air’ to nourishing food, warm clothing, clean water, and regular exercise.^14^

Although naval hygienists often pointed to the universal principles that guided their proposals, they also recognised the distinct features of their sphere of practice. In 1879, Thomas J. Turner, then Medical Director of the American Navy, described the field as one concerned with ‘individuals of only one sex and of limited ages […] whose occupation is pursued under conditions which are [in] almost absolute defiance of all sanitary laws’.^15^ Both naval and military medicine had developed in response to the manpower needs of eighteenth-century conflicts, when deaths from disease outnumbered those from wounds. Armies, like navies, had to deal with diseases exacerbated by crowded living conditions, such as typhus, in addition to those associated with male vice, particularly venereal disease.^16^ Both branches of the military also developed expertise in dealing with ‘diseases of warm climates’ as they were posted around the world, and had to adapt preventive practices to local atmospheric conditions.^17^ Yet, maritime medicine had the added problem of deficiency diseases such as scurvy, and faced the unique challenge of managing health in an isolated environment, with limited space and supplies, over long periods.

Within the closed environment of a naval vessel, two causal categories of disease presented themselves: those relating to the ship, and those to its crew.^18^ The insides of vessels were thought to pose the greatest risk when they were crowded, dirty, wet, and suffused with impure air. Problematic behaviours, on the other hand, included drunkenness, dirtiness, excessive exposure to the wet and cold, imperfect discipline, and overexertion. Significantly, the root causes of illness in both categories were seen as preventable, suggesting the possibility of a perfectly healthy voyage.

The realities of life at sea, however, challenged even the most determined surgeons. Despite the limitless supply of invigorating sea air, the shared living spaces below deck – particularly the forecastle occupied by sailors – were overcrowded, easily dirtied, and difficult to ventilate. Increasingly, the degree to which crews managed to maintain a clean, dry, and airy atmosphere was correlated to their wellbeing. Empirical observation convinced eighteenth-century medical reformers that if similar ships were experiencing different rates of disease in the same area, then the problem must lie somewhere within the afflicted vessels.^19^ In this nascent period of naval hygiene, the chief culprit of any shipboard epidemic was typically identified as noxious air. Miasmatic theory, which linked disease to environmental impurities, was widely accepted by naval medical authorities.^20^ In C.F. Vandeburgh’s 1819 *Mariner’s Medical Guide*, for example, ‘unwholesome effluvias’ were thought to emanate from spoilt cargo, dirty bilge water, and the general humidity and filthiness in the sailors’ living quarters, leading to outbreaks of fever.^21^

However, Vandeburgh also allowed for the possibility of alternate causes, and warned against direct contact with contaminated people or goods. Particularly in the tropics, where contagious diseases raged, exposure to the shore was advised against, and sick individuals were to be immediately isolated and cleaned, their clothes boiled and bedding fumigated.^22^ The Admiralty made similar recommendations to medical officers in 1825, noting that those ‘taken ill of Fever, which [the surgeon] suspects to be infectious’ should be washed in warm water, while their clothes and bedding were boiled and ventilated.^23^ The emphasis on cleanliness and airing that was believed to prevent the outbreak of disease was also considered the best means of checking its spread – whether created internally from foetid airs or transmitted from person to person, the sanitary strategy remained the same.

The early nineteenth century was a period of consolidation in naval medicine, as the recommendations of reformers such as Lind and Blane were implemented.^24^ The ventilation and cleansing of ships was regulated, and personal sanitation was improved by instituting regular washing days and distributing soap. Yet, such practical measures were accompanied by comparatively little theoretical innovation, and eighteenth-century medical texts remained authoritative so long as wind propelled sail. Nonetheless, there was a popular conception in the Victorian era that these opening decades of the century were generally less than healthy. In such accounts, the efforts of medical reformers in curbing the incidence of scurvy and improving provisions merited praise, but with the proviso that sanitary practices made few inroads, with outbreaks of disease ‘justly considered […] as excessive’.^25^ By contrast, Blane’s study of naval medical records between the 1780s and 1820s showed that the number of invalidated men fell by almost two-thirds during this period; a decrease he mainly attributed to the elimination of scurvy and the use of soap, which helped to abolish febrile poisons.^26^ Such estimates aside, the actual impact of such efforts is unclear, as comparative morbidity rates were not routinely tallied until the late 1830s.

The administrator who instituted the collection of medical statistics, William Burnett (1779–1861), took charge of the Navy’s Medical Service in 1838.^27^ His earlier experiences as inspector of hospitals to the Mediterranean Fleet had prompted a publication in 1814 describing that station’s outbreaks of ‘bilious remittent fever’.^28^ Burnett strove to explain diseases based on ‘inferences drawn from nature and truth’ rather than supposition, and robustly defended the prevailing opinion that marsh miasma caused the fever. His second edition explicitly rejected the contagiousness of the fever, but conjectured that the atmosphere around the sick might become vitiated in an unclean ship, ‘and contagion ensue’.^29^ If this form of contingent contagionism had never been observed in practice, it was owing to the Royal Navy’s new commitment to sanitation and discipline. By this time the observance of hygienic practices such as deck-washing was a point of pride, with favourable comparisons being made to the ‘putrid’ state of continental vessels.^30^

The meticulousness with which Burnett studied the Mediterranean fever informed his prevailing approach to naval medicine. While Blane had mandated that surgeons keep patient records, Burnett compiled them into an annual statistical survey of the entire health of the Navy. Information from surgeons’ journals was drawn into tables to reveal sickness and death rates for every station, and across the entire service. Accounts of disease outbreaks were also requested, and surgeons were instructed ‘to trace them to their source […] and explain the means used for destroying the infection’.^31^ Like Burnett, many surgeons would point to local causes (such as marsh gases), as well as shipboard conditions, believing ‘a ship may be said to carry at all times within herself the elements of disease’ (such as foul holds, overcrowding, and dirt).^32^ These views conformed to earliest precepts of Lind, Trotter, and Blane, with an emphasis placed on medical topography and the promotion of cleanliness, dryness, and ventilation as prophylactics. Yet, Burnett’s statistical approach ensured that naval hygiene would henceforth have a quantitative basis. This shift was not only characteristic of medicine’s modernisation in the nineteenth century, but also reflected wider administrative trends, as demographic data was sought to inform policies across state sectors.^33^

The pursuit of medical statistics was only one innovation that marked the transition to the steam age. In the 1840s numerous hygienic texts were published to consolidate practices as preventive methods were perfected.^34^ The conventional wisdom of cleaning, drying, and ventilating a ship as thoroughly as possible became something of an incantation in this period. For the naval surgeon John Wilson, ‘every thing in the life of a sailor’ depended on these three measures: ‘his comfort, health, and happiness’. ^35^ Pointing to numerous instances where diseases had been traced to decomposing organic matter in the ship’s bowels, Wilson maintained that if everything within an afflicted vessel was ‘taken out, washed, white-washed, aired, and dried’, the seeds of sickness would be eradicated.^36^

The link between organic decay and disease migrated into the steam era, but had been seen as particularly threatening under sail. This was because the very material from which such ships were built, wood, was itself an organic substance. Wet, rotting, vegetative matter was closely associated with tropical diseases, as the scent of putrefaction emanated from swamp areas ridden with malaria and yellow fever. Unseasoned or ‘green’ wood was thought to be prone to the same process of putrid decomposition when damp, and its use in naval construction was frequently blamed for epidemics.^37^ Indeed, the belief in constitutionally ‘unhealthy’ ships was associated with the supposedly inherent corruption of their timbers and planks. Building vessels in absolute dryness with aged or treated wood became a priority, but even so, crews were constantly at risk when disease could emanate from the interface of water and wood. Similarly, naval surgeons constantly warned against the wet washing of wooden decks, seeing cleanliness without dryness as almost more perilous to health than not cleansing at all.^38^ Alexander Armstrong, a surgeon who became Director-General of the Naval Medical Department in 1865, believed there was ‘no more fertile source of disease’ than the ‘constant inhalation of a moist atmosphere’ from evaporation off the floors.^39^ His sick list was a litany of scurvy, rheumatism, catarrh, bronchitis, and other pulmonary and intestinal diseases that he attributed primarily to damp. Like many hygienists, Armstrong vociferously advocated for the dry rubbing of the decks with sand to avoid saturation.^40^

Naval hygiene in the mid-century was thus well attuned to conditions under sail. The introduction of ironclads, however, promised to transform many of the parameters of its practice. With metal replacing wood, and engines supplanting sails, maritime medicine faced new problems and potential solutions, albeit with an unwavering certainty that the causes of disease would be conquered through a mastery of the environment.

## 2 Steam-Powered Hygiene

The transition to steam represented the clearest expression of the Victorian Navy’s modern, industrial character, as the engines that had powered Britain’s manufacturing revolution were adapted to ensure its naval supremacy. Following the French use of armoured steamers in the Crimean War, the British heavily invested in the new crafts promising faster speed and improved defences.^41^ The Admiralty’s transition from using wooden sailing ships to its increased investment in armoured, iron, engine-driven vessels in the mid-nineteenth century is often recounted through shifts in technology and naval architecture. Yet, these were also changes that affected life aboard ship. The organisation of naval medicine, with its emphasis on environmental management, had to take into consideration the modified design of modern vessels. Equally, the manpower crisis plaguing the Navy precipitated improvements to seafarers’ welfare in which hygiene was to play a role.^42^

The mechanisation of ships undoubtedly offered the world’s navies an opportunity to transform conditions afloat. During the relative peace of the nineteenth century, there was also a clear willingness amongst the leading naval powers to share their knowledge, and medical experts from Britain, France, Scandinavia, and the United States read and disseminated one another’s findings.^43^ Although all nations faced similar challenges, the French emerged as the undisputed leaders of naval medicine, with several dedicated training schools and a research journal established by the mid-century. As Michael A. Osborne has shown, the French linked naval medicine to their imperial ambitions, and the field’s prestige attracted ambitious modernisers.^44^ Certainly Jean-Baptiste Fonssagrives (1823–1884), who taught naval hygiene at Montpellier, was internationally recognised as the definitive authority of medicine under steam, and both the British and Americans frequently deferred to his findings.^45^ Although British set up medical courses for the Navy at Netley in 1871 at Haslar in 1881 to train surgeons in the rudiments of naval hygiene, the discipline was seen as more practical than innovative until its association with tropical medicine at the close of the century. In the United States, meanwhile, there was a sense of frustration with the slow progress made by naval medicine as a serious discipline, and the American naval medical inspector Albert Gihon complained that surgeons rarely enjoyed the respect and authority of their European counterparts.^46^ Despite their particular admiration for French naval medicine, the British were determined to build on their own traditions of hygiene and discipline to make the most of the transition to steam.

The effective administration of naval medicine necessitated a close adaptation of preventive measures to conditions afloat. For John D. MacDonald (1826–1908), Professor of Naval Hygiene at Netley, medical officers required ‘a competent knowledge of the structure and internal economy of [the] ships’ in which they served.^47^ It also meant that ironclads, with their structural differences to wooden ships, mandated a new approach to naval hygiene. At sea, however, the traditional emphasis on dryness, cleanliness, and proper ventilation remained constant. During the transition from sail to steam, the Admiralty’s medical instructions never wavered on these points.^48^ However, steamers offered new opportunities for attaining these ideals. This section explores how the technological advances offered by steam were employed in the service of hygiene, and how surgeons confronted the material challenges posed by the new vessels.

The problem of ventilating the areas below decks had preoccupied the earliest writers on maritime medicine, and was seen as no less important in the age of steam.^49^ This was not only a matter of providing a breathable atmosphere in enclosed areas, but was also explicitly directed at clearing away the ‘impure’ air that was blamed for many illnesses. Effective ventilation thus entailed two processes: clean air had to be drawn in, and vitiated air circulated out. If either element was flawed, the consequences could be deadly. In 1846, thirteen passengers were suffocated on the transport vessel *Maria Somes*, and two years later a similar fate befell seventy-three immigrants aboard the *Londonderry* when its captain battened down the hatches during a storm.^50^ Such tragedies precipitated investigations into respiration and circulation requirements.

To evade the threat of suffocation, the minimum amount of space that could be allotted to each passenger needed to be specified. To this end, surgeons sent detailed measurements of their ships to the Medical Department for consideration.^51^ However, it was suggested that the cubic footage per head was ultimately less important than ensuring a constant supply of air. According to one authority, it was theoretically possible to ‘live and breathe healthfully in a box no larger than a coffin’ provided ‘a gentle stream of air were kept constantly passing through it’.^52^ The focus therefore turned to the *hourly* requirement of air, a calculation that proved contentious. When a former captain, George S. MacIlwaine, summarised the evidence in 1893, he cited experts whose estimates ranged anywhere between 150 to 3000 cubic feet.^53^ Using the work of renowned physiologist Michael Foster, which included the chemical constitution of expired air, MacIlwaine proposed an hourly rate of 3705 , which was countered by engineers who argued for a far lower figure.^54^

While the sums were up for debate, most commentators agreed that steamships had a greater capacity for ventilation than sailing vessels. This was not due to the actual design of the newer ships, which had far fewer natural openings than their wooden counterparts.^55^ Their considerable advantage was in being able to power artificial ventilation systems. Before the use of engines, only ‘natural’ methods of ventilation could be used – typically involving various combinations of scuttles, hatches, shafts, and cowls, with wind-sails angled against their openings to imperfectly direct air below decks. While a variety of manual fans and bellows had been trialled on wooden vessels, these had been inefficient, labour-intensive devices.^56^ The engines of steamships, on the other hand, could easily drive mechanical fans. Notwithstanding their utility, there was much debate over where the fans should be positioned and whether they should be used primarily for supply or extraction.^57^

Steamships had an additional advantage for producing the currents necessary for ventilation: heat. Applying the principle that hot air rises, steam from the boilers could be redirected throughout the ship to create natural draughts. Supply inlets were added to areas below decks that were linked to heated airshafts, and steam-jet ventilators allowed for a more targeted approach. The noise and engine-dependence of such methods could prove problematic, however.^58^ With so many different areas needing to be ventilated, naval vessels were ultimately fitted with multiple systems that combined both natural and artificial means of supplying air: steam and hand-driven fans were joined by traditional scuttles, pipes, and cowls.^59^ There was little uniformity to these arrangements; the Admiralty’s willingness to trial new schemes meant that numerous methods were concurrently in use in the late nineteenth century. MacDonald’s 1881 textbook on naval hygiene described dozens of ventilation of apparatuses over some hundred pages – a focus justified by the view that fresh air was the ‘best disinfectant’.^60^

The subject of ventilation was also ripe for the scientific thrust of Victorian naval medicine, which employed quantitative analyses of air composition, respiration rates, and ventilators’ capacity to judge competing systems. These studies combined advances in chemistry, physiology, and physics, and showed how complementary concepts in medicine and engineering could improve naval efficiency. It also demonstrated how civilian and military concerns could overlap, with cowl ventilation tests run by the Sanitary Institute in 1878, for example, becoming a focus of naval interest.^61^ Although the link between impure air and disease undoubtedly warranted surgeons’ keen attention to ventilation, it is nonetheless striking that medical officers such as Henry Edmonds mastered the technical skills necessary to modify their ships’ systems to promote better airflow, or that they were given the leeway to do so.^62^ This was possibly because naval architects tended to treat ventilation as an afterthought, laying pipes in odd places, or creating channels between the cabins and bilges that emitted ‘highly objectionable perfumes’.^63^ Medical officers had little choice but to make the best of such arrangements. At the Haslar Naval Medical School, set up in 1881, ventilation was taught at the neighbouring Portsmouth dockyards so that surgeons could understand the inner workings of circulation systems under construction.^64^

Steamships not only had the capacity to produce good air, but also good water. Providing sufficient potable water for long cruises was a long-standing challenge of maritime medicine. The traditional solution of storing water in casks carried a risk of contamination from wood decay, and boiling was recommended to destroy the ‘animalculae’ that bred within them.^65^ The introduction of metal tanks in the early nineteenth century was thus met with enthusiasm, and because the British built them of iron rather than lead, as the French did, they did not encounter similar problems of lead poisoning.^66^ Nonetheless, ships frequently had to stop to refill their limited stores. This was a fraught exercise in a period where communication with foreign shores, particularly in warm climates, was seen as a health hazard. Despite the Admiralty’s efforts to map fresh water sources, surgeons regularly traced outbreaks of stomach and bowel complaints to tainted water from foreign ports, an association strengthened by the mid-century discovery that cholera was waterborne.^67^ By the 1870s, medical officers were supplied with chemical kits to test the quality of water brought aboard.^68^ By this stage, however, many relied on their ships’ capacity to condense water from steam, a process enabled by the introduction of evaporators and condensers.^69^ *Normandy*’s apparatus, patented in 1851, streamlined the process of distilling, aerating, and cooling potable water from steam generated in the boilers. With such breakthroughs, naval vessels no longer had to run the risks associated with making frequent landfall. Although they could never hope to be entirely independent of outside influences, hygienists increasingly saw their ships as self-contained, floating fortresses against the sources of disease.

Engine-driven ships still needed to stop and refuel, however. Coaling stations were rapidly set up to service naval vessels around the globe, and some of the old fears that had attended water collection transferred to coal. In his 1893 treatise on tropical hygiene, Fleet surgeon R.W. Coppinger described coal as a potential vector for transmitting the ‘materies morbi’ of malarial fever to men aboard.^70^ Drawing on his understanding of chemical reactions, Coppinger also warned that coal could undergo oxidation changes in moist tropical weather, potentially producing dangerous ‘marsh gases’ and hydrogen sulphide.^71^ Furthermore, coal was liable to spontaneously combust in the heat, representing a new danger faced by steamships in the tropics. It was not only coal that posed a threat; the conductive metal siding of steamers made their interiors intolerably hot and unhealthy in warm climates.^72^ The vessels’ improved capacity for ventilation would prove vital in offsetting such effects, and particularly benefitted the stokers who worked near the blazing heat of the engines.^73^

Throughout this period there was continued anxiety about the relationship between climate and health that prefigured the development of tropical medicine that would recast these associations in bacteriological terms. Steamships moved more quickly in and out of warm regions than sailing vessels could, and allowed for less time to physically ‘acclimatise’ to the changes in temperature that were seen as a precipitating cause of disease.^74^ Instead, superficial adjustments to clothing and diet were recommended in tropical areas, and exertion was to be avoided during the hottest parts of the day.^75^ It was thus something of an advantage that steamships required less manual labour to operate than sailing vessels.

For naval hygienists, the transition to steam thus offered both new opportunities and challenges for maritime health. Despite the passage of time and a complete transformation of the shipboard environment, the preventive strategy prevailed. Nowhere was this more apparent than in the paradoxical quest for dryness at sea. The hygienic fight against moisture and humidity was far more difficult to wage at sea than on land, with ships described as houses ‘immersed in water’ and exposed to the elements.^76^ Damp was perceived as a distinct health hazard: at best, it exacerbated pulmonary conditions such as catarrh, rheumatism, and bronchitis; at worst, it triggered the rot and decay at the heart of lethal fevers. Miasmas fostered in moist holds and bilges were frequently held responsible for diseases ranging from yellow fever to tuberculosis.^77^ The French hygienist Jean-Baptiste Fonssagrives may have coined the popular aphorism ‘a damp ship is an unhealthy ship’, but he never downplayed the difficulties of ridding a ship of moisture. Indeed, he argued that metal ships were harder to dry than wooden ones, and his American counterpart, Albert Gihon, observed that ‘watery vapour’ remained a scourge of maritime sanitation late into the century.^78^

Given the fears over organic decomposition in wooden ships, steamers ought to have carried less risk of damp-related diseases. Yet, the steel and iron sides of the new vessels could become coated with condensation during temperature changes, leading to the phenomenon of ‘sweating’ walls. One naval surgeon recalled an engineering officer who could ‘scrape up water from the sides of his cabin with the hollow of his hand’, with ‘water running down and wetting everything’.^79^ In merchant vessels, this problem dismayed medical inspectors, who observed that sailors’ bunks and bedding were often wet through as a result of poor environmental management.^80^ When a sanitary commission investigated merchant ships in 1890, they found rheumatic sailors ‘sweltering in the heat of the overcrowded, badly ventilated cabin’, with exposed limbs resting against the cold, wet, iron sides of the ships.^81^ On naval vessels, the Admiralty tried sheathing the decks with non-conductive lining, and trialled substances such as cork, tiles, and cement around the bunks. London’s Port Medical Inspector, William Collingridge, advocated for similar measures aboard merchant ships, where there were fewer provisions made for sailors’ health.^82^ When it came to instituting medical reforms, the civilian and commercial spheres generally followed the Navy’s lead.

The other major problem of moisture – that of foul water accumulating in the bilges – remained intractable in the steam age. The lowest recesses of vessels invariably filled with the runoff from every deck, creating noxious cesspools whose disease-causing properties were likened to ‘a flame in a thatch cottage’.^83^ Keeping the bilges dry was therefore a priority, and allegedly ‘tenfold more important’ on steamers than on wooden ships.^84^ On such vessels, woodchips from the stores wound their way to the ship’s bottom and mixed with coal dust and other organic impurities that ostensibly emitted poisonous ‘marsh gases’ as they decayed. Equally, when seawater and slop water containing such putrescible matter pooled together, traces of iron sulphide, present in the metal framing and plating, interacted with the organic waste to produce toxic gases. While the deadly emissions were initially linked to miasmatic theory, chemical analysis at the close of the nineteenth century identified the mix as hydrogen sulphide, a foul-smelling compound several times more lethal to inhale than carbon monoxide.^85^

Pumping and drying out the bilges was thus seen as a vital matter throughout the steam age. No less important was the routine cleaning of the ship from top to bottom. Indeed, cleanliness was the absolute priority of maritime medicine in the nineteenth century – the *sine qua non* without which sailors’ health was sure to suffer. This belief presupposed the link between dirt and disease, which equally propelled the public health movement in Britain. On land, the focus was on removing nuisances, creating sewers, and whitewashing residences. The Royal Navy had long anticipated these concerns, and had instituted washings and sanitary inspections following the recommendations of Trotter and Blane. Its celebrated reputation for cleanliness was only enhanced by technological innovations following the transition to steam.

The presence of the boilers particularly benefitted personal hygiene. A constant supply of hot water meant that men could look forward to cleanliness as a ‘duty as well as a pleasure’, with a weekly warm wash with soap.^86^ The notion that keeping clean was a ‘duty’ reflected the notion that all seafarers were partially responsible for their own health – and those of their shipmates – through sanitary precautions. The same logic underlay the requisite cleaning of dirty clothing and bedding, which evoked similar hygienic scrutiny given their association with skin complaints ranging from herpes to eczema and ulcers.^87^ Steamers could also breed their own dirt. Machinery affixed to the engine room floors meant ‘accumulations of dirt and other offensive rubbish’ collected beneath them, contaminating the air with unhealthy emissions.^88^ The ‘filth’ of oil and coal dust were also a hazard faced by engineers and stokers, and yellow fever was even attributed to the ‘pools of mud, formed and grease and dirt’ around the engines, which ostensibly emitted foul gases in warm climates.^89^ Chloride of zinc was liberally used to neutralise this oily, organic waste.^90^

The Navy’s emphasis on cleanliness was not only a medical concern, but reflected the broader gentrification of the Victorian sea service. Recruitment shortages spurred efforts to attract more sailors, and conditions and pay improved as a consequence. Maintaining dry, clean, and comfortable vessels formed part of efforts to raise the Navy’s reputation, and spotless decks and personal presentation now appeared as a visible symbol of enhanced naval discipline. Its dedication to cleanliness was consequently pursued for both medical and moral reasons. New technologies helped to maintain the highest sanitary standards. Superheated steam from the exhaust system could be conveyed throughout the ship and used as a powerful disinfectant – a method particularly useful for clearing the filthy forecastles of merchant ships of vermin and cockroaches.^91^ The steam cleaning of clothing and bedding at ports also become a favoured alternative to quarantine, demonstrating the spread of modern methods for checking disease.^92^ As the use of chemical cleansers became ubiquitous at the close of the century, vast quantities of chloride-based disinfectants began to be sprayed throughout newly arrived vessels to ensure their purity.

In fact, chemicals had been used aboard naval ships throughout the transition from sail to steam. Chlorine solutions were poured into bilge water to destroy organic matter, nitrogen dioxide – a strong oxidant – was considered best for yellow fever outbreaks, and bleaches such chloride of lime were dissolved into the air to sanitise the atmosphere. Alternatively, a liberal application of quicklime or Condy’s fluid (potassium permanganate) was used as an all-purpose disinfectant.^93^ The rapidly expanding chemical industry showed the potential for modern, scientific approaches to hygiene, and was enthusiastically embraced by the Navy. Indeed, chemical cleaners became particularly prevalent after the rise of germ theory, but even for more traditional hygienists, a sprinkling of quicklime or chloric acid could mitigate the effects of contingent contagion by ‘sweetening the air’.^94^ If anything, surgeons became worried that an over-reliance on disinfectants would mask the smell of decay.^95^

Many of the developments of naval hygiene in the steam age ultimately involved a straightforward adaptation of new solutions to old problems, reinforcing long-established tropes. Yet, the foundations of naval medicine were not completely static during this period. The relationship between theory and practice forms is examined in the next section, showing how naval surgeons aligned themselves in contemporary debates over the origin and spread of disease.

## 3 Causes and Consequences

Maritime medical texts stressed that ships’ surgeons were in a privileged position to study disease. As they traversed the world, medical officers encountered a wide variety of ailments, and were able to monitor their progress amongst a fixed population in a controlled environment. The Naval Medical Department sought to exploit this unique vantage point by requiring surgeons to keep journals recounting their ships’ medical history and listing every sickness they treated. They were explicitly asked to engage in contemporary debates over the nature of infectious diseases, and to weigh the influence of geography, climate, and human contact as causal agents. This was not meant to be an exercise in idle speculation. According to the scientific spirit of the age, surgeons were expected to record all the atmospheric measurements that might have a bearing on health, and to faithfully monitor every stage of sickness. Armed with increasingly precise meteorological instruments, chemical tests, and statistical methods, medical officers refined naval hygiene for the modern era. Yet, when it came to accounting for the cause of disease, consensus eluded them. Many of the adaptations of steam technology to medical concerns rested on long-standing assumptions about the environmental origins of shipboard epidemics. Increasingly, however, surgeons found these explanations insufficient, pointing to incidences of disease on vessels that were perfectly ‘sweet and clean’. While bacteriological theories offered an alternative etiological model by the century’s close, the sanitary principles underpinning maritime medicine continued to hold sway. Bolstered by evidence that the Navy’s hygienic efforts yielded better health outcomes than those experienced by the less-disciplined Merchant service, cleanliness retained its pre-eminent status across the medical spectrum.

As has been shown, naval medicine emphasised the need for dryness, ventilation, and cleanliness as panaceas against the presumed causes of disease: organic decay and impure air, exacerbated by humidity. Such causes could be endemic to particular regions, or to a ship itself, if the conditions aboard were foetid enough. While factors such as diet emerged as important contributors to sea diseases such as scurvy, air quality was still emphasised in Lind’s account of successfully trialling citrus juice.^96^ Avoiding tropical ports, purifying the air, cleaning the bilges, and drying the decks thus represented a comprehensive strategy for counteracting the chief agents of illness.

Increasingly, specific disease categorisation in the nineteenth century brought more precise attributions of cause: some, such as alcoholism, were linked to vice, while others – particularly respiratory disorders – came from exposure to the cold and damp. Temperance, dietary moderation, and proper clothing were recommended as practical precautions against such maladies. The broad category of fevers and other infectious diseases evaded such straightforward attributions, however: they struck individuals regardless of their habits or station, often afflicted those who spent a night ashore, but also attacked those who never left the ship. It is attempts to account for these diseases that form the focus of the remainder of this article.

The Royal Navy was particularly embroiled in etiological debates because ships had long been recognised as vectors of disease. The rationale of maritime quarantine was the presumed contagiousness of certain illnesses, and the practice of impounding sickly ships was periodically enforced in Britain from the seventeenth century onwards.^97^ By the late eighteenth century, however, opponents to quarantine convincingly argued that the supposedly contagious diseases – yellow fever and plague – had not been imported despite decades of commerce with afflicted regions. The idea that such diseases were limited to certain places, arising from local miasmas, was pitted against the theory that people or goods acted as agents of transmission. For those objecting to it, quarantine was not only medically unnecessary, but also politically oppressive and detrimental to trade.^98^ Opinion particularly wavered over the communicability of yellow fever, dividing those who blamed foreign importation from those who held local insanitary conditions accountable.

The Navy’s position on quarantine and contagion was far from fixed in the Victorian period, and the Admiralty recommended that both sanitary measures and isolation be instituted at the first signs of sickness.^99^ Successive medical directors held considerably different positions on the issue in the nineteenth century, and their views could never hope to influence medical officers posted far from Britain. Accordingly, attitudes amongst naval medical personnel varied in their journals and published accounts of epidemics. What is clear is that the transition to steamers, and the continued emphasis placed on hygiene, did not forestall instances of shipboard diseases.

The fate of the steam-sloop HMS *Eclair* in 1845 became a particular point of contention when two-thirds of its crew died of a presumed yellow fever outbreak on return from the African station. Mark Harrison has described how this disaster figured in debates over contagionism and quarantine, as several crew members died in isolation following the ship’s arrival in Britain.^100^ Official inquiries added little clarity, with investigators reaching different conclusions about whether or not the *Eclair* was carrying yellow fever, and whether it was contagious. The alternative explanation assigned the virulence of the fever to the insanitary conditions aboard the vessel – a position reluctantly endorsed by the Navy’s Medical Director William Burnett. Although the Admiralty significantly improved ventilation systems on ironclads during this period in response, speculation over the *Eclair*’s high mortality continued.

Almost all Victorian naval hygienists scoured the *Eclair* case for evidence supporting their preferred understanding of fevers. However, writings by and for naval surgeons were less concerned with the merits of quarantine than on strategies to evade disease altogether. Former surgeon John Wilson drew from his encounters with African marsh fever to deny its contagiousness, instead insisting that the ‘virus or miasm’ behind the *Eclair*’s fever outbreak ‘was formed and radiated in the ship […] in the limbers, or other parts concealed – the issue of decomposition and emanation, or heat, matter, and moisture’.^101^ Rather than imposing pointless quarantines, ‘cleaning and clearing’ was the only possible defence.^102^ The notion that the *Eclair*’s fever was generated in its filthy recesses gained a following amongst those who saw the entire justification for maritime sanitation embodied in the disaster.^103^ By contrast, Alexander Bryson made a lengthy study of African diseases in 1847, and concluded that the ill-fated crew had contracted the fever from malarious emanations issuing from the banks of the Sherbro River in Sierra Leone.^104^ Contrarily, Gavin Milroy, a medical inspector and editor of the *Medico-Chirurchical Review*, confidently declaimed the *Eclair*’s innate sickliness owing to poor ventilation and ‘other causes of insalubrity’.^105^ A committed sanitarian, he downplayed the extent of medical disagreement by stating that ‘no one can well doubt the cause’ of ‘the exceeding and persistent unhealthiness of such ships as […] the *Eclair*’.^106^ Although the vessel was renamed in 1846, it retained a reputation for disease, showing that the trope of constitutionally unhealthy ships transferred to the steam age – with faulty ventilation taking the place of rotted timbers as a fundamental flaw.

Arguments over the *Eclair* thus became a proxy for wider debates within the medical community about how local conditions related to the propagation of disease. There was little argument that certain localities were unhealthy, with tropical areas seen as particularly lethal.^107^ Yet, it was not entirely clear whether the conditions that caused fevers in such areas could be replicated in the hold of a ship to produce similar diseases. While sailing vessels could potentially generate marsh-like gases in their moist, wooden interiors, steamers defied such easy comparisons. Equally, while filth and decay were still cited as the primary culprits of disease in the steam age, ships’ surgeons often struggled to reconcile sanitarian ideas with their own observations. Their journals state that fevers struck despite their surroundings being ‘very clean and dry’ from daily scrubbing and scraping, or with ‘holds, bilges and every part of the ship as dry and sweet as it is possible to keep them’.^108^ In these cases, external causes, such as contact with pestilential ports or over indulgence, had to be identified. Even in an enclosed naval vessel, there were still myriad possible causal agents. Maintaining a salubrious environment may still have been considered necessary for evading disease, but it was by no means sufficient.

The attribution of filth as a causal factor came under attack during Alexander Bryson’s directorship of the Naval Medical Department from 1864 to 1869. Under his watch, the annual compiling of surgeons’ journals into the service’s official health report became an exercise in editorialising about the ‘true’ causes of epidemics. Bryson had complained that the Navy’s data collection efforts were insufficient as early as 1856, when he edited his first report. The requirement that surgeons describe meteorological and hygienic conditions when epidemics struck, he wrote, unfairly favoured environmental explanations of disease. Instead, surgeons should ‘distinctly state whether the persons first attacked had or had not been exposed to infection or contagion, or whether they had or had not visited any port, place or ship’ in the weeks preceding an outbreak.^109^ Bryson’s commitment to contagionism principally related to two diseases: yellow fever and cholera, which he insisted were not spontaneously generated aboard ship. In subsequent years he constantly returned to this theme, warning against contact with both diseases, and contradicting surgeons who believed they originated in foul bilges. In the *Report* for 1858, Bryson explained how the HMS *Icarus* had communicated yellow fever from Belize to Jamaica, carrying the disease from an infected region to a healthy one.^110^ In 1861, he critiqued surgeons who claimed that yellow fever and cholera stemmed from bilgy holds, leading to futile cleaning efforts.^111^ Once Bryson became Director-General of the Medical Department, his colleague Alexander Mackay continued to edit the *Reports* from a contagionist perspective, correcting surgeons who blamed yellow fever on atmospheric conditions and ascribing outbreaks to contact with the disease ashore instead.^112^ In one case, Mackay dismissed the ‘absolutely worthless’ observations that had been supplied, the result of ‘fanciful’ sanitarian theories that blamed ‘accumulations of every abomination, defective drainage, crowding, squalor and poverty, heat and humidity’ for yellow fever whether such conditions were present or not.^113^ Every case of yellow fever, he countered, could be traced to contact with a sick individual.

Bryson espoused an altogether more scientific approach to naval medicine. Central to this vision was the collection of data, which he described in his essay on ‘Medicine and Medical Statistics’ for the Navy’s 1849 *Manual of Scientific Enquiry*.^114^ The Navy’s new scientific spirit was exemplified by this volume’s emphasis on methodical observation across disciplines. As Michael S. Reidy has argued, Naval authorities in this period actively solicited scientific expertise to overcome long-standing navigational, oceanographic, and meteorological problems.^115^ In particular, Humboltian scientific methodology, such as the mapping of natural phenomena and the collection of quantitative data, were embraced to ensure Britain ‘ruled the waves’. For Bryson, this meant that surgeons could use hygrometric and thermometric instruments to chart atmospheric conditions on health, and measure pulse and respiration rates in different climates. In places associated with disease, topographical notes would uncover sources of ‘noxious effluvia’.^116^ Equally, by comparing the ship’s movements and outbreaks of disease, one might determine ‘to an hour […] the exact period of incubation in certain endemic and contagious diseases’.^117^ For Bryson, scientific methods and instruments would help isolate the sources of disease, separating those that were local or constitutional from ones that were clearly contagious. While the optimism of sanitarians stemmed from their belief that a perfectly clean ship would be perfectly healthy, for Bryson the real possibility of reducing mortality from infectious diseases would come from studying their methods of transmission.^118^

Bryson put his ideas into practice as the Navy’s Medical Director. The aforementioned *Reports* became more quantitative, with detailed mortality and morbidity tables provided to enable comparative, geographic analysis.^119^ The annual publication of these statistics allowed health reformers to trace improvements over time, as rates of sickness were mapped out in recurring categories each year. When T. Graham Balfour analysed the medical returns of the Army and Navy in 1872, he confidently demonstrated the ‘marked reduction’ in naval deaths in the 1860s compared to the 1830s, which had dropped from an average of 8.8 to 6.35 per 1000.^120^ Balfour also suggested that the very act of publishing medical data had spurred sanitary reforms, which were more aggressively pursued in the years following the issuing of the first *Report* in the late 1830s.^121^ The dawn of the steam age clearly coincided with a new era of scientific investigation and analysis that ensured higher standards of health aboard the new ironclads.

In fact, the *Eclair* episode had demonstrated that steamers still experienced dramatic outbreaks of disease, and there was evidence suggesting that fevers were particularly virulent on the new ships. It was suggested that the engines exacerbated overcrowding and reduced air quality, given the disproportionate amount of space they occupied below decks.^122^ Further, their heat was thought to generate a harmful quasi-tropical ambiance that promoted the spread of zymotic diseases. As vessels increased in size and ventilation methods improved, steamers were progressively considered more conducive to health. In other regards, the steam era heralded unequivocal medical advantages. Engine-driven ships were faster than wooden ones, leading to fewer nutritional deficiencies and a more rapid transfer of the sick to hospitals.^123^ It also meant that there was less time to acclimatise to changes in heat and humidity, a concern as such transitions were seen as constitutionally damaging.^124^ In compensation, however, quitting unhealthy localities was quickly accomplished when needed – an important provision for those who believed that malaria and cholera were endemic to tropical regions.

If an evidence-based approach indicated that naval health was gradually improving, it did little to resolve debates around disease causation. In 1841, the statistician A.M. Tulloch was surprised to learn that there were comparatively few cases of fever in South America despite the prevalence of warm, wet, marshy conditions.^125^ He hoped that the Navy’s medical data would help to dispel false theories linking diseases to particular environments, instead offering insight into the ‘specific agencies to which the absence or presence of particular diseases is attributable’.^126^ Far from reassuring commentators that their particular etiological theory was correct, data collection often raised more questions than it answered. When Robert Armstrong, a deputy inspector of the fleet, examined the Navy’s reports in 1843, he too noted that the presence of swamps had little correlation with outbreaks of disease.^127^ He conducted his own chemical tests on the air, water, and soil in the Dutch East Indies for signs of the ‘Batavian fever’ then raging, and found no evidence of noxious gas. With scant evidence for the theory of ‘marsh miasmas’ caused by ‘vegeto-animal’ poisons, Armstrong suggested that medical officers’ unfounded beliefs blinded them to alternative explanations for disease, such as the ‘electrical condition’ of the air.^128^

Certainly, naval hygiene texts had always been ambivalent about the causes of common sea diseases. Lind had seen scurvy as a problem of both diet and air quality, and a century later it was still associated with both causes, with cold and damp added as aggravating factors.^129^ When the Admiralty appointed a committee to investigate the severe outbreak of scurvy that had hampered its 1875 arctic expedition, its detailed final report attributed the initial outbreak to the ‘absence of lime juice’ amongst the sledding parties, but also implicated the ‘comparatively vitiated’ atmosphere of the lower decks, as well as the damp and extreme temperature changes.^130^ The state of medical uncertainty in this era invariably led to such oscillations. On its own, nutrition gained some recognition as an important factor in seafarers’ wellbeing, with the Medical Department acknowledging in the *Health Report* for 1867 that ‘without a proper diet’, cleanliness, dryness, and ventilation would ‘prove unavailing to preserve health and prevent disease’.^131^ While dietary adjustments were made in 1859, 1861, and 1879 to align the caloric intake of sailors with their workload (with stokers given extra rum and sugar), the actual benefits of these changes continued to be conflated with other preventive measures.^132^ Rather than supplant old ideas, new theories came to coexist alongside them: on one page it was possible to see cholera attributed to contaminated water, on another to dietary excesses.^133^ Likewise, it was possible to find medical authorities claiming that cool air draughts were both beneficial and detrimental to health.^134^ Naval hygiene was rarely presented as a systematic science, but as a series of recommendations based on practice and observation. If one measure did not work; another could always be tried. Despite the haphazardness of this approach, it allowed for a more holistic approach to health, with personal habits, the atmosphere, and sanitation all requiring consideration.

Notwithstanding the flexibility of hygienic recommendations, ventilation and sanitation were still the favoured methods of preserving health. The link between cleanliness and wellness was particularly intractable because dirt and disease were so visibly linked in the nineteenth century. Medical authorities frequently ascribed the Navy’s unhealthy past to the grime and squalor that had prevailed before the mid-eighteenth century. With a rigorous emphasis on dryness, cleanliness, and ventilation, steamships were deemed two to three times more efficient than sailing vessels.^135^ Moreover, the Navy could always favourably compare itself to the Merchant service: although morbidity rates were not tallied for the latter, mortality rates were almost twice as high by 1899 (9.6 per 1000 compared to the Navy’s 4.9).^136^ Given the poor sanitary conditions aboard merchant vessels, it was widely assumed that the Navy’s success at environmental management had optimised the health of its crews.^137^ Certainly the Medical Department endorsed this view, as the Director-Generals of the late-Victorian era credited the steady decline in deaths from disease almost entirely to ‘the success of sanitary efforts’.^138^

## 4 Conclusion

From its inception in the eighteenth century, until the close of the nineteenth century, cleanliness, dryness, and ventilation reigned as the holy trinity of naval hygiene. Improvements in sailors’ health during this period were continuously ascribed to these factors. When Walter Reid addressed surgeons at the Naval Medical School in 1889, he stressed that the safety of the British Empire depended on their ability to maintain the lives of British seamen.^139^ Nothing was more crucial to this objective than preventive medicine. By this stage, the conversion to steam had been fully effected and the great *Majestic* class of pre-Dreadnoughts was replacing the older ironclads. No expense was spared in the building of these elaborate vessels, and their design incorporated all the hygienic advances of the steam era, from artificial ventilation systems, to water condensers, to washing machines to facilitate the cleaning of clothes and bedding. As steamship design came to be standardised, hygienic principles were incorporated into naval architecture. Medical officers also came to enjoy a medical education tailored to the designs of these ships.

Naval surgeons were also increasingly trained in the laboratory. As bacteriology gained acceptance, it proved particularly instrumental to the development of tropical medicine – a specialism long associated with the Navy as ‘diseases of warm climates’ were an occupational hazard of seafarers. It is no coincidence that Patrick Manson, the ‘father of tropical medicine’, worked at various Seamen’s Hospitals in London while shaping ideas about parasitology and the spread of malaria.^140^ Naval medicine was thus well situated to embrace germ theory, which offered a clear explanation for why cleaning and ventilating had been comparatively effective at minimising sickness at sea.

For naval hygienists, germ theory may not have appeared particularly revolutionary. The idea that organic impurities spread disease was reasonably widespread in the Victorian period, and microbes could be easily substituted for the nebulous ‘poisonous matter’ that had been traditionally blamed for shipboard epidemics. For John McDonald, the notion of airborne bacteria merely justified the need to perfect ventilation, adding a new twist to an old, established principle.^141^ Efforts at limiting the spread of recognised vectors, such as mosquitos, further enhance the effectiveness of hygienic arrangements in later years. Ships’ surgeons simply had to reframe old practices within a new framework that still prioritised prevention.

Although Victorian hygienists debated the contagiousness of certain diseases and how to evade them, nineteenth-century naval medicine did not significantly depart from its founding precepts. Instead of using their contained environment to test etiological theories, surgeons were persuaded to perfect hygiene practices instead. This emphasis contributed to naval discipline and order, allotting surgeons a role in regulating life afloat. Yet while hygienic principles did not radically shift during the transition from sail to steam, new technologies were still harnessed to improve medical efficiency. The process of adapting engine-driven ships to serve the welfare of seafarers showed the positive effects of investing in health – not in the complete eradication of disease, but in the improved capacity to evade its probable causes.

## Footnotes

1.
Norman Chevers, *On the Preservation of the Health of Seamen* (Calcutta: Military Orphans Press, 1864), 18.

2.
James Lind, *An Essay on the Most Effectual Means of Preserving the Health of Seamen* *in the Royal Navy* (London: 1757); Gilbert Blane, *Observations on the Diseases Incident to Seamen* (London: Joseph Cooper, 1785); Thomas Trotter, *Medicina Nautica* (London: T. Cadell and W Davies, 1797).

3.
Vaughan Myles Dutton, *Mortality Decline in the Royal Navy, 1850–99* (unpublished DPhil thesis: University of Oxford, 2009). Dutton argues that the decline in naval mortality and morbidity rates were due to dietary changes and sanitary measures.

4.
Anthony S. Wohl, *Endangered Lives: Public Health in Victorian Britain* (London: Methuen, 1983); Christopher Hamlin, *Public Health and Social Justice in the Age of Chadwick* (Cambridge: Cambridge University Press, 1998); and F.B. Smith, *The People’s Health, 1830–1910* (London: Croom Helm, 1980).

5.
As David McLean has shown, naval authorities sometimes faced opposition on land, as during the 1848–9 Plymouth cholera outbreak. David McLean, *Public Health and Politics in the Age of Reform: Cholera, the State and the Royal Navy in Victorian Britain* (London: Taurus, 2006).

6.
Mark Harrison, ‘Medicine and the Management of Modern Warfare’, *History of Science*, 34 (1996), 379–410; Roger Cooter and Steve Sturdy, ‘Of War, medicine and modernity’, in Roger Cooter, Mark Harrison and Steve Sturdy (eds), *War, Medicine and Modernity* (Stroud: Sutton, 1998), 1–21.10.1177/00732753960340040111618782

7.
Gilbert Blane, *A Short Account of the Most Effectual Means of Preserving the Health of Seamen* (London, 1780), 4.

8.
Katherine Foxhall, *Health, Medicine and the Sea: Australian Voyages, c. 1815–60* (Manchester: Manchester University Press, 2012); Tim Carter, *Merchant Seamen’s Health, 1860–1960: Medicine, Technology, Shipowners and the State in Britain* (Woodbridge: The Boydell Press, 2014).

9.
Admiralty, *Regulations and Instructions for the Medical Officers of His Majesty’s Fleet* (London: W. Clowes and Sons, 1825), 13.

10.
Michael Brown, ‘From Foetid Air to Filth: The Cultural Transformation of British Epidemiological Thought, c. 1780–1848’ *Bulletin of the History of Medicine*, 82 (2008), 515–44; Christopher Hamlin, ‘Providence and Putrefaction: Victorian Sanitarians and the Natural Theology of Health and Disease’, *Victorian Studies*, 28 (1985), 381–411.

11.
The first statistical report on ‘the Health of the Navy’ was published in 1840, and annual editions appeared from 1857.

12.
Gilbert Blane, *A Brief Statement of the Progressive Improvement of the Health of the Royal Navy* (London: W. Nicol, 1830), 28.

13.
John Wilson, *Outlines of Naval Surgery* (Edinburgh: Maclachlan, Steward & Co., 1846), 34.

14.
John D. MacDonald, *Outlines of Naval Hygiene* (London: Smith, Elder, & Co., 1881), 2.

15.
Thomas J. Turner, ‘Hygiene of the naval and merchant marine’, in Albert H. Buck (ed.), *A Treatise on Hygiene and Public Health*, Vol. II (London: Sampson Low *et al.*, 1879), 177.

16.
The controversial Contagious Diseases Acts of the 1860s, which enforced the medical examination of prostitutes, were specifically designed to curb venereal disease in the military. See Judith R. Walkowitz, *Prostitution and Victorian Society: Women, Class, and the State* (Cambridge: Cambridge University Press, 1980), 67–148.

17.
Pratik Chakrabarti, *Medicine and Empire, 1600–1960* (Basingstoke: Palgrave Macmillan, 2014), 40–56.

18.
T. Spencer Wells, *The Scale of Medicines With Which Merchant Vessels Are To Be Furnished*, 2nd edn (London: John Churchill, 1861), 17; William Collingridge, ‘Health in the marine service’, in Thomas Oliver (ed.), *Dangerous Trades: The Historical, Social, and Legal Aspects of Industrial Occupations as Affecting Health* (London: John Murray, 1902), 184; Wilson, *op. cit.* (note 13), 35.

19.
Blane, *op. cit.* (note 7), 4.

20.
Christopher Hamlin, ‘Predisposing Causes and Public Health in Early Nineteenth-Century Medical Thought’, *Social History of Medicine*, 5 (1992), 43–70; John V. Pickstone, ‘Dearth, dirt, and fever epidemics: rewriting the history of British public health, 1780–1850’, in Terrance Ranger and Paul Slack (eds), *Epidemics and Ideas: Essays on the Historical Perception of Pestilence* (Cambridge: Cambridge University Press, 1992), 125–48.10.1093/shm/5.1.4311612776

21.
C.F. Vandeburgh, *The Mariner’s Medical Guide* (London: Baldwin, Cradock, and Joy, 1819), 26–37.

22.

*Ibid.*, 39–40.

23.
Admiralty, *op. cit.* (note 9), 13.

24.
R.S. Allison, *Sea Diseases: The Story of a Great Natural Experiment in Preventive Medicine in the Royal Navy* (London: John Bale, 1943), 189–90.

25.
Gavin Milroy, *The Health of the Royal Navy Considered* (London: Robert Hardwicke, 1862), 5.

26.
Blane estimated that 1 in 3.3 naval men were invalidated in 1782, compared to 1 in 8.9 by 1829. Blane,*op. cit.* (note 12), 11–14.

27.
Christopher Lloyd and Jack L.S. Coulter, *Medicine and the Navy, 1200–1900: Volume IV, 1815–1900* (Edinburgh: E. & S. Livingstone Ltd, 1963), 3–5.

28.
William Burnett, *A Practical Account of the Fever Commonly Called the Bilious Remittent* (London: J. Callow, 1814).

29.
William Burnett, *A Practical Account of the Mediterranean Fever* (London: J. Callow, 1816), 17.

30.
Kevin Brown, *Poxed and Scurvied: The Story of Sickness and Health at Sea* (Barnsley: Seaforth, 2011), 102–103.

31.
Admiralty, *The Queen’s Regulations and the Admiralty Instructions for the Government of Her Majesty’s Naval Service* (London: Her Majesty’s Stationery Office, 1862), 397.

32.
Journal of Surgeon John Jack, *HMS Liffey*, 1859–60, ADM 101/205, TNA.

33.
T. Graham Balfour, ‘Comparative Health of Seamen and Soldiers, as Shown by the Naval and Military Statistical Reports’, *Journal of the Statistical Society of London*, 35 (1872), 1–24; William Farr, ‘The Application of Statistics to Naval and Military Matters’, *Journal of the Royal United Services Institution*, 3 (1859), 209–24.

34.
Robert Armstrong, *The Influence of Climate and Other Agents on the Human Constitution, with Reference to the Causes and Prevention of Disease Among Seamen* (London: Longman, Brown, Green, and Longman, 1843); F.F. Sankey, *Familiar Instructions in Medicine and Surgery with Observations on the Means of Maintaining the Health of Men on Ship Board* (London: Parker, Furnivall, and Parker, 1846); Frederick James Brown, *Questions and Observations in Hygiene* (London: John Churchill, 1849).

35.
Wilson, *op. cit.* (note 13), 46.

36.

*Ibid.*, 94.

37.
For example, Gavin Milroy attributed fever outbreaks aboard the *Herald* (1856) and *Hecate* (1858) to wood rot; Milroy, *op. cit.* (note 25), 26. See also Alexander Bryson, *Report on the Climate and Principal Disease of the African Station* (London: William Clowes and Sons, 1847), p. 229; Wells, *op. cit.* (note 18), 38.

38.
See e.g. Robert Finlayson, *An Essay on the Baneful Influence of So Frequently Washing Decks* (London: T. Egerton, 1823).

39.
Alexander Armstrong, *Observations on Naval Hygiene and Scurvy* (London: John Churchill, 1858), 86–7.

40.
Bryson, *op. cit.* (note 37), 221.

41.
Peter Padfield, *Rule Britannia: The Victorian and Edwardian Navy* (London: Routledge, 1981); Eric J. Grove, *The Royal Navy Since 1815: A Short History* (Basingstoke: Palgrave Macmillan, 2005).

42.
Eugene L. Rasor, *Reform in the Royal Navy: A Social History of the Lower Deck, 1850–1880* (Hamden: Archon Books, 1976).

43.
Netley professor John D. MacDonald recommended the steam cleaning methods of the Swedish naval hygienist Abraham Eklund, for example, and Staff Surgeon John Buckley produced a translation of work by Leroy de Mericourt, Medical Director of the French Navy. See MacDonald, *op. cit.* (note 14), 116; Leroy de Mericourt, *Modern Naval Hygiene*, John Buckley (trans.) (London: Griffin & Co., 1875).

44.
Michael A. Osborne, *The Emergence of Tropical Medicine in France* (Chicago: The University of Chicago Press), 111–53.

45.
See e.g. Turner, *op. cit.* (note 15), 194; MacDonald, *op. cit*. (note 14), 162.

46.
Albert L. Gihon, *Practical Suggestions in Naval Hygiene* (Washington: Government Printing Office, 1873), 2.

47.
MacDonald, *op. cit.* (note 14), 9.

48.
Admiralty, *op. cit.* (note 31), 389.

49.
Arnold Zuckerman, ‘Disease and Ventilation in the Royal Navy: The Woodenship Years’, *Eighteenth-Century Life*, 11 (1987), 77–89.11616926

50.

*The Times* (Friday, 8 December 1848), 4.

51.
Journal of Surgeon John Buckley, HMS *Rinaldo*, 1870, ADM 101/182, TNA.

52.
Henry Edmonds, ‘Ventilation of Iron-Clad Ships of War’, *Journal of the Royal United Service Institution*, 9 (1866), 313.

53.
George S. MacIlwaine, ‘Ventilation of Ships’, *The Journal of the Royal United Service Institution*, 35 (1893), 2–3.

54.

*Ibid.*, 12.

55.
MacDonald, *op. cit.* (note 14), 70.

56.

*Ibid.*, 77–86.

57.
Se e.g. John D. MacDonald, ‘On Ship Ventilation as a Development of Naval Hygiene’, *Journal of the Royal United Service Institution*39 (1895), 721–732; and ‘On the Ventilation of Ships’, *Journal of the Royal United Service Institution* 18 (1875), 136–51.

58.
R.W. Coppinger, ‘Sanitation Afloat’, in C. E. Shelley (ed.), *Transactions of the Seventh International Congress of Hygiene and Demography. Volume III. Section VIII: Naval and Military Hygiene* (London: Eyre and Spottiswoode, 1892), 28.

59.
See John Taylor’s description of ventilation aboard the *Sappho*and *Scylla* in MacIlwaine, *op. cit.* (note 53), 15.

60.
MacDonald, *op. cit.* (note 14), 50.

61.
The privately-financed cowl tests were subsequently challenged, although they were cited by naval hygienists. P. F. Nursey, *The Kew Cowl Tests* (London: Hickson, Ward and Co., 1902).

62.
Edmonds received permission to implement an exhaust system that expelled spent air via shafts connected to the steamer’s main funnel. Journal of Surgeon H. Edmonds, *HMS St Jean D’Acre*, 1859, ADM 101/203, TNA.

63.
Journal of Surgeon David Lloyd Morgan, *HMS Royal Alfred*, 1867, ADM 101/232, TNA.

64.
Walter Reid, ‘The Duties of Naval Surgeons’, *BMJ* (23 March 1889), 645–6.10.1136/bmj.1.1473.644PMC215518720752641

65.
See Vandeburgh, *op. cit.* (note 21), 29; Wilson, *op. cit.* (note 13), 41.

66.
Mericourt, *op. cit.* (note 43), v; Chevers, *op. cit.* (note 1), 4–5; Gihon, *op. cit.* (note 46), 90.

67.
William Saunders, *Hygienic, Medical and Surgical Hints for Young Officers in the Royal and of the Merchant Navy* (London: John Churchill, 1856), 18–20.

68.
Admiralty, *The Queen’s Regulations and Admiralty Instructions for the Government of Her Majesty’s Navy* (London: Her Majesty’s Stationary Office, 1879), 333.

69.
Wells, *op. cit.* (note 18), 54.

70.
R.W. Coppinger, ‘Tropical naval hygiene’, in Andrew Davidson (ed.), *Diseases of Warm Climates* (Edinburgh: Young J. Pentland, 1893), 83. For a discussion of how cargo was perceived as a vector of disease, see David S. Barnes, ‘Cargo, “Infection”, and the Logic of Quarantine in the Nineteenth Century’, *Bulletin of the History of Medicine*, 88 (2014), 91–5.10.1353/bhm.2014.001824769803

71.
Coppinger, *ibid.*, 83.

72.
Joseph Wilson, *Naval Hygiene* (Washington: Government Printing Office, 1870), 19–20.

73.
For a discussion of stokers’ health as it related to high temperature exposure, see Turner, *op. cit.* (note 15), 188–92.

74.
Armstrong, *op. cit.* (note 34), 27–32.

75.
James Bird, ‘The Means of Practically Applying the Principles of Medical Geography, for the Preservation of the Health of Soldiers and Seamen in Foreign Climates’, *Journal of the United Service Institution*, 1 (1 January 1858), 223–34.

76.
Coppinger, *op. cit.* (note 70), 81.

77.
Bird, *op. cit.* (note 75), 223, 234.

78.
Jean-Baptiste Fonssagrives, *Traité d’hygiène navale* (Paris: J.B. Baillière, 1856), 261; Albert L. Gihon, ‘The Need for Sanitary Reform in Ship-Life’, *Public Health Papers and Reports*, 3 (1876), 89.PMC227212319599962

79.
Edmonds, *op. cit.* (note 52), 326.

80.
Collingridge, *op. cit.* (note 18), 182–3; Henry E. Armstrong, ‘The Hygiene of Merchant Ships, with Especial Regard to Seamen’, *Public Health*, 7 (1894), 64.

81.
Lancet Special Sanitary Commission, ‘Report on Sanitation at Sea’, *The Lancet* (20 September 1890), 635.

82.
William Collingridge, ‘Practical Points in the Hygiene of Ships and Quarantine’, *The Lancet* (5 May 1894), 1111.

83.
Chevers, *op. cit.* (note 1), 23.

84.
Turner, *op. cit.* (note 15), 180.

85.

*Ibid.*, 181; MacDonald, *op. cit.* (note 14), 245; Coppinger, *op. cit.* (note 70), 82.

86.

*Statistical Report of the Health of the Navy, for the year 1864* (London: House of Commons, 1867), 18. See also Brown, *op. cit.* (note 34), 24.

87.
Wilson, op. cit. (note 13), 35; Wells, *op. cit.* (note 18), 61.

88.
Milroy, *op. cit.* (note 25), 31–2.

89.
Wells, *op. cit.* (note 18), 39; Wilson, *op. cit*. (note 72), 12, 175.

90.
See e.g. Saunders, *op. cit.* (note 67), 18; Wells, *op. cit.* (note 18), 40.

91.
Archibald Finlay, *The Seaman’s Medical Guide*, 2nd edn (London: George Philip & Son, 1891), 24.

92.
Frederick Montizambert, ‘Modern quarantine in Canada and the United States’, in C.E. Shelley (ed.), *op. cit.* (note 58), 11–12.

93.
See e.g. Turner, *op. cit.* (note 15), 200; MacDonald, *op. cit.* (note 14), 248–51.

94.
Finlay, *op. cit.* (note 91), 23.

95.
Journal of Staff Surgeon Thomas Browne, *HMS Griffon*, 1878, ADM 101/235, TNA.

96.
James Lind, *A Treatise on Scurvy in Three Parts* (Edinburgh: Sands, Murray and Cochrane, 1753), 97.

97.
John Booker, *Maritime Quarantine: The British Experience, c. 1650–1900* (Aldershot: Ashgate, 2007).

98.
Mark Harrison, *Disease and the Modern World: 1500 to the Present Day* (Cambridge: Polity, 2004), 97–105.

99.
Admiralty, *Regulations and Instructions for the Medical Officers of His Majesty’s Fleet* (London: W. Clowes and Sons, 1835), 24.

100.
Mark Harrison, *Contagion: How Commerce has Spread Disease* (New Haven: Yale University Press, 2012), 80–106.

101.
Wilson, *op. cit.* (note 13), 82.

102.

*Ibid.,*91.

103.
See e.g. Edmonds, *op. cit.* (note 52), 319; Gihon, *op. cit.* (note 46), 113.

104.
Bryson, *op. cit.* (note 37), 179–94.

105.
Milroy, *op. cit.* (note 25), 19.

106.

*Ibid.,*53.

107.
On the geography of disease and theories of acclimatisation, see e.g. Philip D. Curtin, ‘The White Man’s Grave: Image and Reality, 1750–1850’, *Journal of British Studies*, 1 (1961), 94–110; David Livingstone, ‘Human Acclimatisation: Perspectives on a Contested Field of Inquiry in Science, Medicine, and Geography’, *History of Science*, 25 (1987), 359–94.10.1177/00732753870250040211621496

108.

*Statistical Report of the Health of the Navy, For the Year 1861* (London: House of Commons, 1864), 63; *Statistical Report of the Health of the Navy, For the Year 1862* (London: House of Commons, 1865), 3.

109.

*Statistical Report of the Health of the Royal Navy, For the Year 1856* (London: House of Commons, 1858), iii–iv.

110.

*Statistical Report of the Health of the Royal Navy, For the Year 1858* (London: House of Commons, 1861), iv–v.

111.

*Statistical Report of the Health of the Royal Navy, For the Year 1860* (London: House of Commons, 1863), iv.

112.

*Statistical Report 1862, op. cit.* (note 108), 102.

113.

*Statistical Report of the Health of the Royal Navy, For the Year 1864* (London: House of Commons, 1867), 90–1.

114.
Alexander Bryson, ‘Medicine and medical statistics’, in J.F.W. Herschel (ed.), *A Manual of Scientific Enquiry Prepared for Use of Her Majesty’s Navy* (London: John Murray, 1849), 445–64.

115.
Michael S. Reidy, *Tides of History: Ocean Science and Her Majesty’s Navy* (Chicago: University of Chicago Press, 2008).

116.
Bryson, *op. cit.* (note 114), 452.

117.

*Ibid.*, 458.

118.
Alexander Bryson, ‘Health of the Navy for 1860’, *Annals of Military and Naval Surgery and Tropical Medicine and Hygiene*, 1 (1863), 16.

119.

*A Statistical Return of the Health of the Royal Navy for the Year 1859* (London: House of Commons, 1862), iii.

120.
Balfour, *op. cit.* (note 33), 5.

121.

*Ibid.,*18.

122.
Milroy, *op. cit.* (note 25), 31.

123.
MacDonald, *op. cit.* (note 14), 235.

124.
Collingridge, *op. cit.* (note 18), 183.

125.
A.M. Tulloch, ‘Comparison of the Sickness, Mortality, and Prevailing Diseases Among Seamen and Soldiers’, *Journal of the Statistical Society of London*, 4 (1841), 15–16.

126.

*Ibid.*, 16.

127.
Armstrong, *op. cit.* (note 34), 47–53.

128.

*Ibid.,*74–6.

129.
Armstrong, *op. cit*. (note 39), 32.

130.
Admiralty, *Report of the Committee Appointed by the Commissions of the Admiralty to Enquire Into the Causes of the Outbreak of Scurvy in the Recent Arctic Expedition* (London: Her Majesty’s Stationery Office, 1877), iii.

131.

*Statistical Report of the Health of the Royal Navy, For the Year 1867* (London: House of Commons, 1869), 13.

132.
For an overview of the probable effects of naval dietary changes on the overall health of British seafarers in the nineteenth century, see Dutton, *op. cit*. (note 3), 168–201.

133.
Saunders, *op. cit.* (note 67), 19, 22.

134.
MacDonald, *op. cit.* (note 14), 53.

135.
William A. Guy, *Public Health: A Population Introduction to Sanitary Science* (London: Henry Renshaw, 1870), 163.

136.
Collingridge, *op. cit.* (note 18), 184.

137.
See the *BMJ*’s five-part ‘Report on the Hygienic Condition of the Mercantile Marine’, beginning in *BMJ* (12 January 1867), 36–7.

138.
Mortality fell from 12 to 5 per 1000 between 1856 to 1886. *Statistical Report of the Health of the Navy, For the Year 1887* (London: House of Commons, 1888), xviii.

139.
Reid, *op. cit.* (note 64), 645.

140.
Michael Worboys, ‘The emergence of tropical medicine: a study in the establishment of a scientific specialty’, in Gerard Lemaine (ed.), *Perspectives on the Emergence of Scientific Disciplines* (The Hague: De Gruyter, 1976), 83.

141.
MacDonald, *op. cit.* (note 14), 49.

